# RESUME: Turning an SWI acquisition into a fast qMRI protocol

**DOI:** 10.1371/journal.pone.0189933

**Published:** 2017-12-20

**Authors:** Serena Monti, Pasquale Borrelli, Enrico Tedeschi, Sirio Cocozza, Giuseppe Palma

**Affiliations:** 1 IRCCS SDN, Naples, Italy; 2 Department of Advanced Biomedical Sciences, University “Federico II”, Naples, Italy; 3 Institute of Biostructures and Bioimaging, National Research Council, Naples, Italy; Henry Ford Health System, UNITED STATES

## Abstract

Susceptibility Weighted Imaging (SWI) is a common MRI technique that exploits the magnetic susceptibility differences between the tissues to provide valuable image contrasts, both in research and clinical contexts. However, despite its increased clinical use, SWI is not intrinsically suitable for quantitation purposes. Conversely, quantitative Magnetic Resonance Imaging (qMRI) provides a way to disentangle the sources of common MR image contrasts (*e*.*g*. proton density, *T*_1_, etc.) and to measure physical parameters intrinsically related to tissue microstructure. Unfortunately, the poor signal-to-noise ratio and resolution, coupled with the long imaging time of most qMRI strategies, have hindered the integration of quantitative imaging into clinical protocols. Here we present the RElaxometry and SUsceptibility Mapping Expedient (RESUME) to show that the standard acquisition leading to a clinical SWI dataset can be easily turned into a thorough qMRI protocol at the cost of a further 50% of the SWI scan time. The *R*_1_, $R_{2}^{*}$, proton density and magnetic susceptibility maps provided by the RESUME scheme alongside the SWI reconstruction exhibit high reproducibility and accuracy, and a submillimeter resolution is proven to be compatible with a total scan time of 7 minutes.

## Introduction

In the last two decades Susceptibility Weighted Imaging (SWI) [1] has been increasingly used in neuroimaging MRI protocols, owing to its ability to detect the presence of paramagnetic (deoxyhaemoglobin, haemosiderin, ferritin, etc.) or diamagnetic (calcium hydroxyapatite or apatitelike minerals, myelin, oxyhaemoglobin, etc.) compounds [2–5].

Numerous studies have demonstrated the clinical relevance of this technique across a wide range of pathological conditions [6], including neuroinflammation, neurodegeneration and head trauma. Moreover, it is valuable in the detection of cerebral micro-bleeds in aging population, in the follow-up of hemorrhagic infarctions, and in the assessment of cavernous malformations, venous thrombosis, and calcium or iron deposition, as thoroughly reviewed in [7–9].

Although several MRI Gradient Echo (GRE) signals (Steady-State Free-Precession—SSFP [10], Echo-Shifted—ES [11], etc.) acquired with different spatial encoding strategies (Projection Acquisition—PA [12], Periodically Rotated Overlapping ParallEL Lines with Enhanced Reconstruction—PROPELLER [13], Echo Planar Imaging—EPI [14], etc.) can be processed to obtain an SWI dataset, it is common practice to rely on a standard 3D single spoiled GRE with a relatively long echo time (usually *T*_*E*_ ∼ 20 ms at 3 T [15]). Such choice, however, seems suboptimal, as at least one more echo at short *T*_*E*_ could fit within an otherwise dead-time of the sequence. Indeed, even at moderately low receiver bandwidths (BW of the order of 100 Hz/pixel), the sampling time of an echo *t*_*s*_ = 1/BW is of the order of 10 ms, thus allowing for the acquisition of one more signal at *T*_*E*_ ∼ 8 ms. The rationale behind the acquisition of more echoes should not be found in their availability without inducing major changes that may alter the original GRE signal at long *T*_*E*_ (in terms of contrast, SNR, etc.). Indeed, multi-echo GRE allows for $R_{2}^{*}$ quantification [16], along with a more accurate estimate of Quantitative Susceptibility Mapping (QSM) compared to what obtained with the single-echo acquisition [17]. These quantitative parameters provide a robust and comprehensive characterization of the tissue susceptibility, which may shed light in many areas of clinical interest (from mineral metabolism [18] to vein segmentation [19, 20]).

On the other hand, a more thorough approach to quantitative MRI (qMRI) is desirable in a wide spectrum of clinical conditions [21], since *R*_1_ and Proton Density (PD) give complementary and more detailed information on water tissue content. These parameters were shown to provide unique insights into the status of white matter in normal aging and demyelinating disease [22–24] or into tumor characterization [25, 26]. In [27], PD was used to derive Macromolecular Tissue Volume Fraction (MTVF) maps, which provide a sensitive measure of the disease status in multiple sclerosis patients, and, together with *R*_1_ maps, allow for an estimation of the apparent volume of interacting water protons (strictly related to the properties of local physico-chemical environment). Moreover, *R*_1_, coupled to $R_{2}^{*}$, is a key to determine the stage of haemorrhages [28] or to obtain absolute measures of contrast agent concentrations within tissues [29, 30]. In addition, the joint availability of *R*_1_, $R_{2}^{*}$ and susceptibility measures in Fabry’s disease has been recently exploited to redefine the pathogenetic mechanisms and the incidence of the pulvinar sign in that metabolic disorder [31].

*R*_1_ and PD maps are usually derived from two or more spoiled GRE sequences acquired at fixed *T*_*R*_ and variable Flip Angle (FA) [32]. Therefore, one may consider to append one more GRE sequence (*e*.*g*. with a lower FA) to that used for SWI, QSM and $R_{2}^{*}$ mapping [16]. However, an unpleasant and straightforward consequence of *R*_1_ mapping by variable FAs consists in the increased total acquisition time, which clearly doubles the original duration of the SWI protocol, and certainly limits its clinical applicability.

Here we present the RElaxometry and SUsceptibility Mapping Expedient (RESUME) that halves the duration of the supplementary acquisition, still providing the complete set of qMRI maps (*R*_1_, $R_{2}^{*}$, PD and QSM) along with the SWI processing.

## Materials and methods

RESUME is a qMRI scheme consisting of a 3D acquisition protocol coupled with the analytical solution of the Bloch equations associated to the acquired MRI signals. The protocol is derived from a standard SWI acquisition, modified to sample at least two echoes, and by the addition of one more spoiled GRE sequence with halved repetition time.

First, the analytical solution of the qMRI problem is given for a single-echo spoiled GRE (with repetition time *T*_*R*,0_, echo time *T*_*E*,0_ and FA *θ*_1_) and a multi-echo spoiled GRE (with repetition time 2 ⋅ *T*_*R*,0_, echo times {*T*_*E*, *i*_} and FA *θ*_2_). Finally, more details on the actual acquisition setup will be given.

### Analytical solution of the qMRI equations

#### Spoiled GRE signal

The complex signal of a spoiled GRE sequence is

$$\begin{array}{c} S=K\cdot M_{0}\cdot \text{sin}\theta \cdot \frac{1-E_{1}}{1-E_{1}\text{cos}\theta }\cdot e^{-T_{E}\cdot (R_{2}^{*}+i\gamma_{n}\Delta B)+i\phi_{0}}\;, \end{array}$$ (1)

where *K* is the complex coil sensitivity, *M*_0_ is the equilibrium magnetization, *E*_1_ ≡ exp(−*T*_*R*_ ⋅ *R*_1_), *γ*_n_ is the gyromagnetic ratio of the imaged nucleus, Δ*B* is the local magnetic field variation and *ϕ*_0_ is the phase-shift induced by the RF-pulse [16].

#### ${\text{R}}_{2}^{*}$ map

If *s*_*i*_ represents the magnitude of the *i*-th echo of the multi-echo spoiled GRE, from Eq 1 it follows that

$$\begin{array}{c} \text{log}\;s_{i}=k_{0}-T_{E,i}\cdot R_{2}^{*} \end{array}$$ (2)

for

$$\begin{array}{c} k_{0}=\text{log}\;[\left|K\right|\cdot M_{0}\cdot \text{sin}\theta_{2}\cdot \frac{1-E_{1}}{1-E_{1}\text{cos}\theta_{2}}]\;. \end{array}$$ (3)

Therefore, a simple regression via weighted least squares leads to the following estimates of

$$\begin{array}{c} R_{2}^{*}=-\frac{\sum_{i=1}^{n}w_{i}\left(T_{E,i}-{\bar{T}}_{E}\right)\left(\text{log}\;s_{i}-\bar{L}\right)}{\sum_{i=1}^{n}w_{i}{\left(T_{E,i}-{\bar{T}}_{E}\right)}^{2}} \end{array}$$ (4)

and

$$\begin{array}{c} k_{0}=\bar{L}+R_{2}^{*}\cdot {\bar{T}}_{E}\;, \end{array}$$ (5)

where

$$\begin{array}{c} {\bar{T}}_{E}=\frac{\sum_{i=1}^{n}w_{i}T_{E,i}}{\sum_{i=1}^{n}w_{i}}\;,\;\bar{L}=\frac{\sum_{i=1}^{n}w_{i}\;\text{log}\;s_{i}}{\sum_{i=1}^{n}w_{i}}\;,\;\text{and}\;w_{i}=\frac{1}{{\text{BW}}_{i}\cdot s_{i}^{2}}\;. \end{array}$$ (6)

Eq 4 reduces to

$$\begin{array}{c} R_{2}^{*}=\frac{\text{log}\;(s_{1}/s_{2})}{T_{E,2}-T_{E,1}} \end{array}$$ (7)

in case of double-echo acquisitions.

#### R_1_ map

Eqs 1, 4 and 5 allow the estimation of the signal magnitude at *T*_*E*_ = 0 of the single-echo (*S*_1_) and multi-echo (*S*_2_) spoiled GREs as

$$\begin{array}{c} S_{1}=s_{0}\cdot e^{T_{E,0}\cdot R_{2}^{*}} \end{array}$$ (8)

(*s*_0_ being the magnitude of the single-echo GRE) and

$$\begin{array}{c} S_{2}=e^{k_{0}} \end{array}$$ (9)

(see Eq 3).

On the other hand,

$$\begin{array}{c} S_{1}=\left|K\right|\cdot M_{0}\cdot \text{sin}\theta_{1}\cdot \frac{1-E_{1,0}}{1-E_{1,0}\text{cos}\theta_{1}} \end{array}$$ (10)

and

$$\begin{array}{c} S_{2}=\left|K\right|\cdot M_{0}\cdot \text{sin}\theta_{2}\cdot \frac{1-E_{1,0}^{2}}{1-E_{1,0}^{2}\text{cos}\theta_{2}}\;, \end{array}$$ (11)

where *E*_1,0_ ≡ exp(−*T*_*R*,0_ ⋅ *R*_1_).

Therefore, defining

$$\begin{array}{c} q=S_{1}/S_{2} \end{array}$$ (12)

and

$$\begin{array}{c} k=\frac{\text{sin}\theta_{1}}{\text{sin}\theta_{2}}\;, \end{array}$$ (13)

from Eqs 10 and 11 it comes out that

$$\begin{array}{c} q=\frac{k(1-E_{1,0}^{2}\text{cos}\theta_{2})}{\left(1+E_{1,0}\right)\left(1-E_{1,0}\text{cos}\theta_{1}\right)}\;. \end{array}$$ (14)

Solving with respect to *E*_1,0_, two solutions are found:

$$\begin{array}{c} E_{1,0}^{\pm }=\frac{R\pm \sqrt{R^{2}+4C\left(q-k\right)}}{2C}\;, \end{array}$$ (15)

where

$$\begin{array}{c} R=q(1-\text{cos}\theta_{1}) \end{array}$$ (16)

and

$$\begin{array}{c} C=q\text{cos}\theta_{1}-k\text{cos}\theta_{2}\;. \end{array}$$ (17)

**Fig 1:**
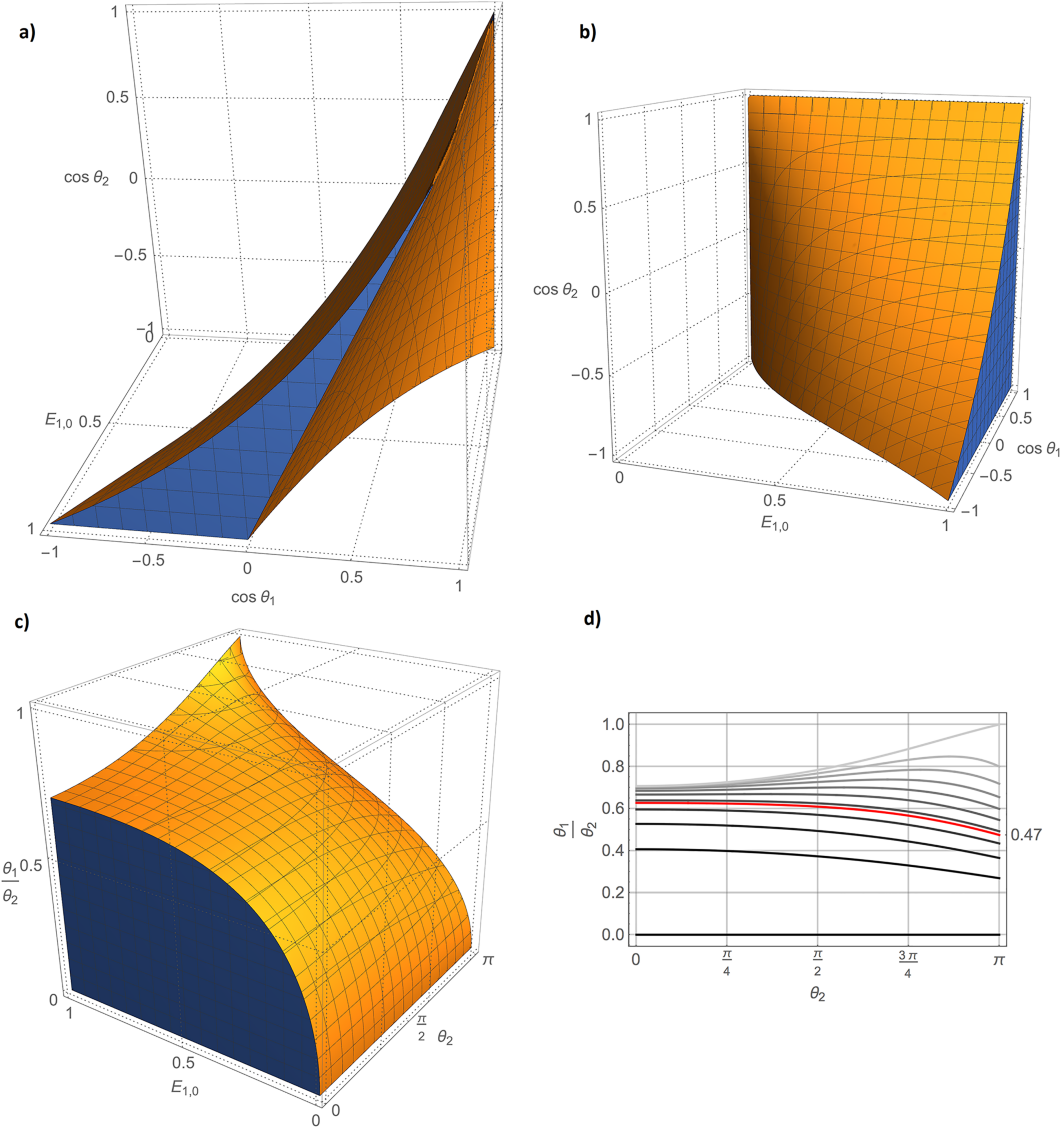
Numerical details about the analytical solutions $E_{1,0}^{\pm }$. a) Region in the parameter space (cos*θ*_1_, cos*θ*_2_, *E*_1,0_) in which both solutions $E_{1,0}^{\pm }$ satisfy the physical constrain 0 < *E*_1_ < 1. b) Region in the parameter space (cos*θ*_1_, cos*θ*_2_, *E*_1,0_) in which solution $E_{1,0}^{+}$ applies. c) Region in the parameter space (*θ*_1_/*θ*_2_, *θ*_2_, *E*_1,0_) in which solution $E_{1,0}^{+}$ applies. d) Family of curves parametrized by *E*_1,0_ (ranging from 0 [black] to 1 [light gray] in step of 0.1) that pose an upper limit to *θ*_1_/*θ*_2_ for $E_{1,0}^{+}$ to be acceptable. The red curve corresponds to *E*_1,0_ = *e*^−1^ and entirely lies in the half-plane *θ*_1_/*θ*_2_ > 0.47.

Unfortunately, for some pairs (*θ*_1_, *θ*_2_) both solutions in Eq 15 may satisfy 0 < *E*_1,0_ < 1 (see Fig 1a)), thus precluding the possibility to find an unambiguous *R*_1_ value for each (*θ*_1_, *θ*_2_, *S*_1_, *S*_2_).

In order to determine which of the solutions in Eq 15 is physically relevant, $E_{1,0}^{+}$ is recognized as

$$\begin{array}{c} E_{1,0}^{+}=E_{1,0}^{+}[\theta_{1},\theta_{2},S_{1}\left(E_{1,0},\theta_{1}\right),S_{2}\left(E_{1,0},\theta_{2}\right)]=f[\theta_{1},\theta_{2},E_{1,0}]\;. \end{array}$$ (18)

Looking for the parametric domain in which the condition

$$\begin{array}{c} f[\theta_{1},\theta_{2},E_{1,0}]=E_{1,0} \end{array}$$ (19)

is satisfied, after some algebraic manipulation, it results that:

$$\begin{array}{c} E_{1,0}=\{\begin{array}{cc} E_{1,0}^{+} & \text{if}\;\text{cos}\theta_{1}\geq \frac{1+\text{cos}\theta_{2}\cdot E_{1,0}\left(2+E_{1,0}\right)}{1+E_{1,0}\left(2+\text{cos}\theta_{2}\cdot E_{1,0}\right)}\;;\\ E_{1,0}^{-} & \text{otherwise}. \end{array} \end{array}$$ (20)

Of note, the condition in Eq 20 explicitly depends on the unknown *E*_1,0_ (see Fig 1b)). However, the graphics in Fig 1c) and 1d) show that for *E*_1,0_ > *e*^−1^ (corresponding to the realistic condition in which *T*_*R*,0_ < *T*_1_), for any 0 < *θ*_2_ < *π* and 0 < *θ*_1_ < 0.47 ⋅ *θ*_2_, $E_{1,0}^{+}$ provides the correct solution to Eq 14. Therefore, in the following it will be assumed that

$$\begin{array}{c} R_{1}=-\frac{\text{log}\;E_{1,0}^{+}}{T_{R}}\;. \end{array}$$ (21)

A thorough analysis of the FAs *θ*_1_ and *θ*_2_ that optimize the *R*_1_ map SNR in the general case of a multi-echo GRE with multiple echoes and variable BW_*i*_ is quite complex and, probably, not really insightful. However, in the specific case of a double-echo GRE acquired with *T*_*E*,1_ = *T*_*E*,0_ and BW_1_ = BW_0_ (which is the most realistic condition—see the section dedicated to acquisition), a useful numerical approach can be adopted. From BW_1_ = BW_0_, it follows that *S*_1_ and *S*_2_ share the same noise power $\sigma_{S}^{2}$. Therefore the variance of the *R*_1_ estimate can be expressed as

$$\begin{array}{cc} \sigma_{R_{1}}^{2}\left(\theta_{1},\theta_{2},R_{1}\right)= & ({\left\{\frac{\partial E_{1,0}^{+}}{\partial S_{1}}\left[\theta_{1},\theta_{2},S_{1}\left(\theta_{1},R_{1}\right),S_{2}\left(\theta_{2},R_{1}\right)\right]\right\}}^{2}+\\ & {\left\{\frac{\partial E_{1,0}^{+}}{\partial S_{2}}\left[\theta_{1},\theta_{2},S_{1}\left(\theta_{1},R_{1}\right),S_{2}\left(\theta_{2},R_{1}\right)\right]\right\}}^{2})\;\cdot \;{\left[\frac{\sigma_{S}}{T_{R,0}\cdot E_{1,0}\left(R_{1}\right)}\right]}^{2}. \end{array}$$ (22)

**Fig 2:**
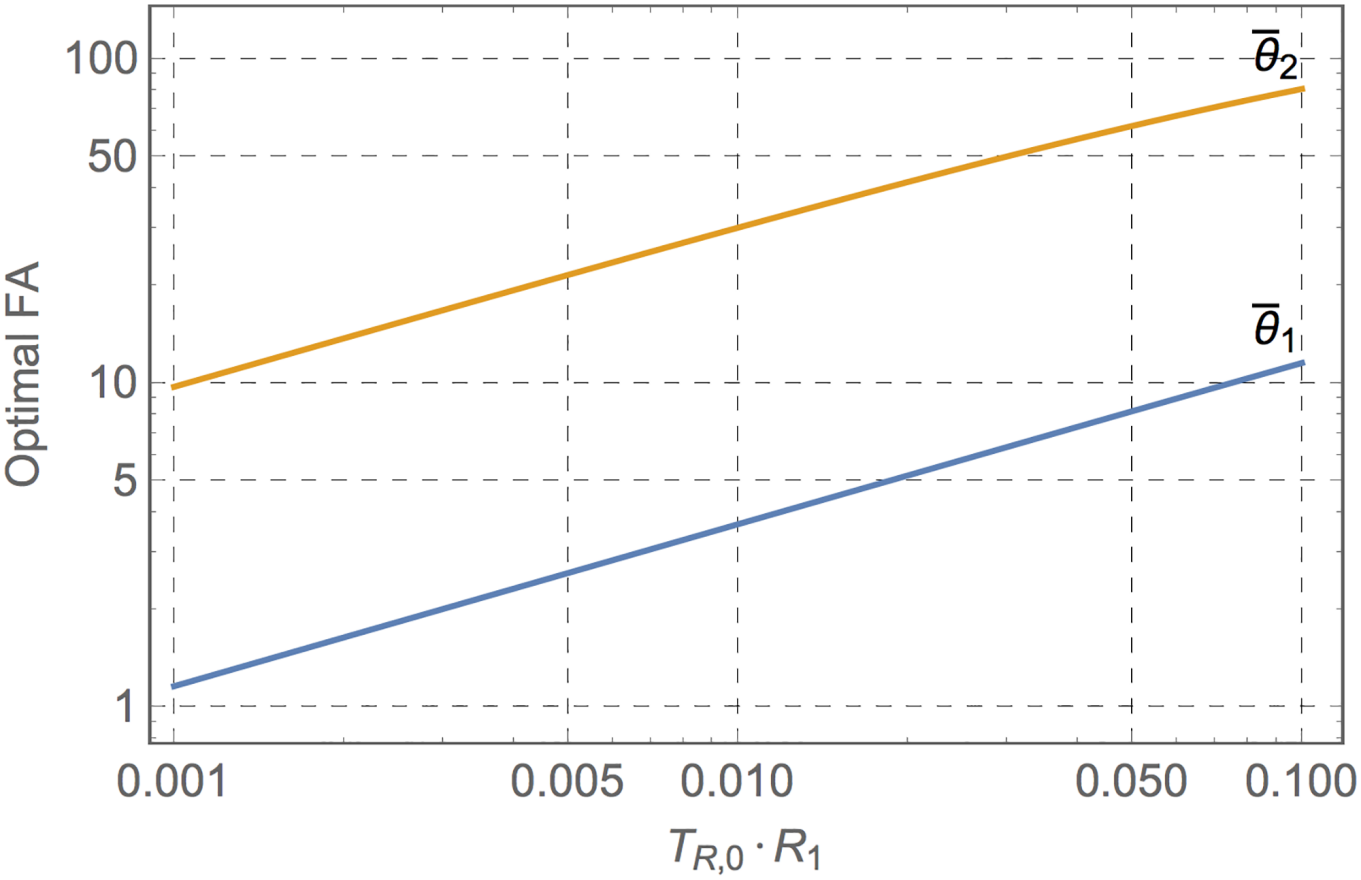
Optimal Flip Angles (FAs) for *R*_1_ mapping. FAs ${\bar{\theta }}_{1}$ and ${\bar{\theta }}_{2}$ of single- and double-GRE sequences that maximize the accuracy of *R*_1_ estimate as a function of the expected *T*_*R*,0_ ⋅ *R*_1_.

The optimal FAs ${\bar{\theta }}_{1}$ and ${\bar{\theta }}_{2}$ are thus obtained by minimizing $\sigma_{R_{1}}^{2}$ for a given *R*_1_; the numerical results are shown for a wide range of practical *R*_1_ ⋅ *T*_*R*,0_ in Fig 2.

#### PD map

From Eq 11, PD can be estimated as

$$\begin{array}{c} \left|K\right|M_{0}=S_{2}\cdot \frac{1-E_{1,0}^{2}\text{cos}\theta_{2}}{\text{sin}\theta_{2}\cdot \left(1-E_{1,0}^{2}\right)}\;. \end{array}$$ (23)

#### QSM

There are plenty of techniques that can be used to derive the QSM from the complex signal in Eq 1 (for a recent overview, see [33]), each more or less recommended depending on the specific application. A highly accurate reconstruction scheme is given by the algorithm introduced in [34], which is here adopted to obtain the map shown in the Results from the phase images of the multi-echo GRE sequences.

### MRI acquisition

MRI was performed using a 3 T scanner (Trio, Siemens Medical Systems, Erlangen, Germany) with a volume transmitter coil and an 8-channel head receiver coil. Sequences were acquired after having obtained an informed consent for experimentation with human subjects. The “Carlo Romano” ethics committee for biomedical activities of “Federico II” University of Naples (Italy) specifically approved the study and the written informed consent form, which was signed by the subject undergoing the MR scan.

The 3D RESUME protocol actually acquired for this study consists of:

1. a single-echo flow-compensated spoiled GRE sequence (Repetition Time: *T*_*R*_ = 14 ms; Echo Time: *T*_*E*1_ = 7.63 ms; FA: *θ*_1_ = 2°);
2. a dual-echo flow-compensated spoiled GRE sequence (Repetition Time: 2 ⋅ *T*_*R*_ = 28 ms; Echo Times: *T*_*E*1_ = 7.63 ms and *T*_*E*2_ = 22.14 ms; FA: *θ*_2_ = 20°).

Both sequences shared the same geometry (pseudo-axial orientation along the anterior commissure-posterior commissure; Field of View (FOV) = 230 × 194 × 166 mm^3^; voxel size Δ*V* = 0.65 × 0.65 × 1.3 mm^3^; GRAPPA acceleration factor = 2) and were acquired with a BW of 190 Hz/pixel in a total acquisition time of 7’ 9”. For each echo, a magnitude/phase reconstruction was enabled, thus obtaining a total of 3 complex volume datasets.

**Fig 3:**
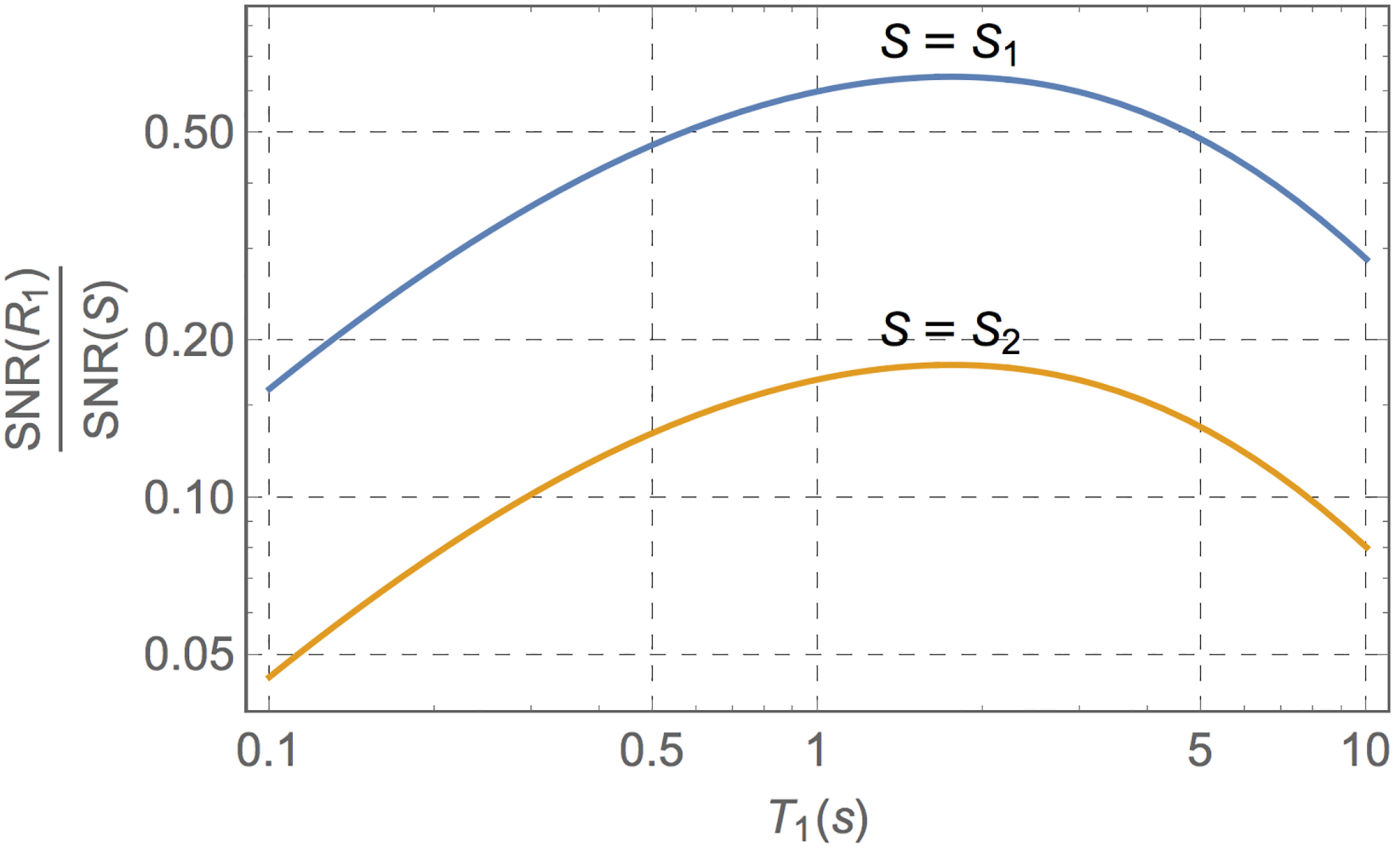
Ratio between the SNR of the estimated *R*_1_ map and the SNR of the *S* images in an average brain parenchyma (with *T*_1,par_ = 1 s) as a function of the expected *T*_1_, for *θ*_1_ = 2° and *θ*_2_ = 20°.

According to the chosen FAs, the SNR of the resulting *R*_1_ map can be estimated from Eq 22. This quality index can be made independent from the noise power within the acquired images by normalizing it to the SNR of the input GRE images (possibly after some denoising) computed in an average brain parenchyma (with a nominal *T*_1,par_ = 1 s). The result is shown in Fig 3 as a function of the expected *T*_1_.

### Denoising

Before computing the $R_{2}^{*}$, *R*_1_ and PD maps according to Eqs 4, 21 and 23, the acquired datasets were denoised following a revised version of the Multi-spectral Non-Local Means (MNLM) approach first described in [16].

Briefly, in this context, *M* different datasets are considered as a multi-component image $X:R^{N}\to R^{M}$ (*N* = 3) with bounded support $\Omega \subset R^{N}$. The MNLM is, then, a class of endomorphisms of the image space, each identified by 3 parameters (*κ*, *ρ* and *ς*), that act as follows:

$$\begin{array}{c} {\left[{\text{MNLM}}_{\kappa ,\rho ,\varsigma }\left(X\right)\right]}_{m}\left(\vec{x}\right)=Y_{m}\left(\vec{x}\right)=\frac{\int_{B_{r}\left[\vec{x}\right]}W_{m}\left(\vec{x},\vec{y}\right)X_{m}\left(\vec{y}\right)d\vec{y}}{\int_{B_{r}\left[\vec{x}\right]}W_{m}\left(\vec{x},\vec{y}\right)d\vec{y}}. \end{array}$$ (24)

In Eq 24 $B_{r}[\vec{x}]$ is the ball centered in $\vec{x}$, whose radius *r* is defined such that

$$\begin{array}{c} \int_{B_{r}[\vec{x}]}W_{m}\left(\vec{x},\vec{y}\right)d\vec{y}=\kappa \;, \end{array}$$ (25)

where

$$\begin{array}{c} W_{m}\left(\vec{x},\vec{y}\right)\equiv \text{exp}[-\frac{d_{\rho }^{2}\left(\vec{x},\vec{y}\right)}{\varsigma^{2}}\cdot \frac{Q_{m}\left(\vec{x}\right)}{\sum_{l=1}^{M}Q_{l}\left(\vec{x}\right)}]\;, \end{array}$$ (26)

$$\begin{array}{c} d_{\rho }^{2}\left(\vec{x},\vec{y}\right)\equiv \sum_{m=1}^{M}\left\{\int_{{\mathbb{R}}^{N}}\frac{{\left|X_{m}\left(\vec{x}+\vec{t}\right)-X_{m}\left(\vec{y}+\vec{t}\right)\right|}^{2}}{\sigma_{m}^{2}\left(\vec{x}\right)+\sigma_{m}^{2}\left(\vec{y}\right)}\cdot \frac{\text{exp}-\frac{{\left\|\vec{t}\right\|}^{2}}{2\rho^{2}}}{{\left(\sqrt{2\pi }\cdot \rho \right)}^{N}}d\vec{t}\right\}, \end{array}$$ (27)

$$\begin{array}{c} Q_{m}\left(\vec{x}\right)\equiv \frac{\int_{\Omega }X_{m}^{2}\left(\vec{y}\right)d\vec{y}}{\int_{\Omega }\sigma_{m}^{2}\left(\vec{x}\right)d\vec{y}}\;, \end{array}$$ (28)

*κ* ∼ 10^2^ ⋅ Δ*V* provides a criterion to dynamically allocate a search window, $\rho ∼\sqrt[{N}]{5\cdot \Delta V}$ is the similarity radius, *ς* ∼ 1 is a dimensionless constant to be manually tuned that determines the filter strength, and $\sigma_{m}\left(\vec{x}\right)$ is the standard deviation of noise of the *m*^th^ image component at $\vec{x}\in \Omega$ (the noise power maps were estimated following [35]).

The denoising of the magnitude datasets (*s*_0_, *s*_1_ and *s*_2_) required by $R_{2}^{*}$, *R*_1_ and PD calculation was performed by exploiting also the information contained within the QSM derived from the phase images. Therefore, a total of *M* = 4 real datasets were jointly processed in Eq 24.

Due to the high computational complexity of the above scheme, a multi-Graphics Processing Unit (GPU) implementation of the MNLM algorithm [36] was adapted to Eq 24 and exploited for fast image processing.

### Field inhomogeneity correction

$B_{1}^{\pm }$ non-ideal profiles critically influence the accuracy of the *R*_1_ and PD estimates. In particular, while *R*_1_ just depends only on $B_{1}^{+}$ through the presence of *θ*_1,2_ in Eq 15 (the receiver coil sensitivity $B_{1}^{-}$ is eliminated in the ratio of signal intensities in Eq 12), PD is also affected by $B_{1}^{-}$ via *S*_2_ in Eq 23.

If a protocol for a fast measurement of *B*_1_ profiles of slab-selective RF-pulses is available on the scanner, the actual *B*_1_ maps can be directly used in Eqs 15 and 23 to obtain bias-free *R*_1_ and PD maps.

In the results shown below, to get rid of such biases, the effective approach based on the information content of the derived maps proposed in [16] was adopted.

### RESUME assessment

The reproducibility and the accuracy of the RESUME maps were estimated on a pool of 10 subjects (5 patients and 5 healthy controls—HCs—were recruited in order to test the method across a broad range of tissue parameters) by applying the following procedure on each of them (hereafter identified by the index *j* = 1, …, 10).

First, to obtain the confidence interval of the RESUME maps, each sequence of the protocol was acquired twice during the same session. Given the redundant set of complex datasets, an ensemble of 2^3^ (cardinality of the Cartesian product of magnitude datasets *s*_0_, *s*_1_ and *s*_2_) × 2^2^ (cardinality of the Cartesian product of phase datasets from the dual-echo GRE—used for QSM) choices of different complete RESUME protocols was produced. Thirty-two different estimates of the relaxometry and susceptibility maps were thus derived and used to estimate the overall reproducibility (accounting for image uncorrelated noise, variations in signal amplification of the MR scanner, tissue temperature fluctuations, etc.) via voxelwise mean ($\mu_{j}\left(\vec{x}\right)$) and standard deviation ($\sigma_{j}\left(\vec{x}\right)$) maps.

Moreover, the RESUME *R*_1_, $R_{2}^{*}$ and PD maps were compared with the corresponding maps derived on the same subject using another qMRI approach established in [16]. This approach is based on two or more multi-echo spoiled 3D GRE sequences, in which all acquisition parameters are kept constant, except the FA, which is varied in order to provide different *T*_1_-weights. The acquired signals are then conveniently combined in order to derive high SNR maps of *R*_1_, $R_{2}^{*}$, PD and magnetic susceptibility.

On the other hand, the RESUME susceptibility map was compared to the QSM derived from an 8-echo GRE sequence (Repetition time: 28 ms; Echo Times: {*T*_*E*, *i*_ = [3.38 + (*i*−1) ⋅ 2.82] ms}; FA: 20°; BW: 400 Hz/pixel), acquired with bipolar readout gradients (no flow-compensation) and the same geometry of the RESUME sequences.

The accuracy was finally evaluated via the difference magnitude ($\delta_{j}\left(\vec{x}\right)$) between RESUME maps and the maps used for comparison.

For evaluation purposes, a healthy control (HC1) was chosen as the common spatial reference for the pool of subjects. The reproducibility ($\left\{\sigma_{j}\left(\vec{x}\right)\;|\;j=2,\ldots ,10\right\}$) and accuracy ($\left\{\delta_{j}\left(\vec{x}\right)\;|\;j=2,\ldots ,10\right\}$) maps obtained for each other subject were, then, spatially normalized to HC1 according to the deformation field given by the elastic registration of the dual GRE image acquired at *T*_*E*,1_ on the corresponding HC1 image. Furthermore, for a numerical assessment of the maps, the median values within the brain mask were extracted for each patient from $\left\{\sigma_{j}\left(\vec{x}\right)\right\}$ and $\left\{\delta_{j}\left(\vec{x}\right)\right\}$.

To compare the RESUME measures with the values found in the literature, an expert neuroradiologist manually drew a set of bilateral Regions of Interest (ROIs) on the *R*_1_ map of HCs in the white matter, in the cortical grey matter, in the head of the caudate nucleus and in the putamen; the mean values of *R*_1_, $R_{2}^{*}$, PD and QSM were then extracted for further comparisons.

Full access to the obtained results is provided at https://figshare.com/s/6d467faa11579c7c0c02.

### Image processing

All data processing, except the QSM (which was obtained by the free tool described in [34]), was performed using an in-house developed library for Matlab (MATLAB^®^ Release 2012b, The MathWorks, Inc., Natick, MA, USA), partly described in previous works [16, 35–37], on a commercial workstation (Intel^®^ Core^™^ i7-3820 CPU @ 3.6 GHz; RAM 16 GB) equipped with 2 GPU boards (NVIDIA GeForce^®^ GTX 690). The demonstrative application of qMRI for vein segmentation, and Oxygen Extraction Fraction (OEF) and MTVF mapping was respectively provided by the MAVEN algorithm [20] and the in-house implementations of the methods described in [27, 38].

## Results

Each RESUME processing took 10 minutes on the above described workstation.

**Fig 4:**
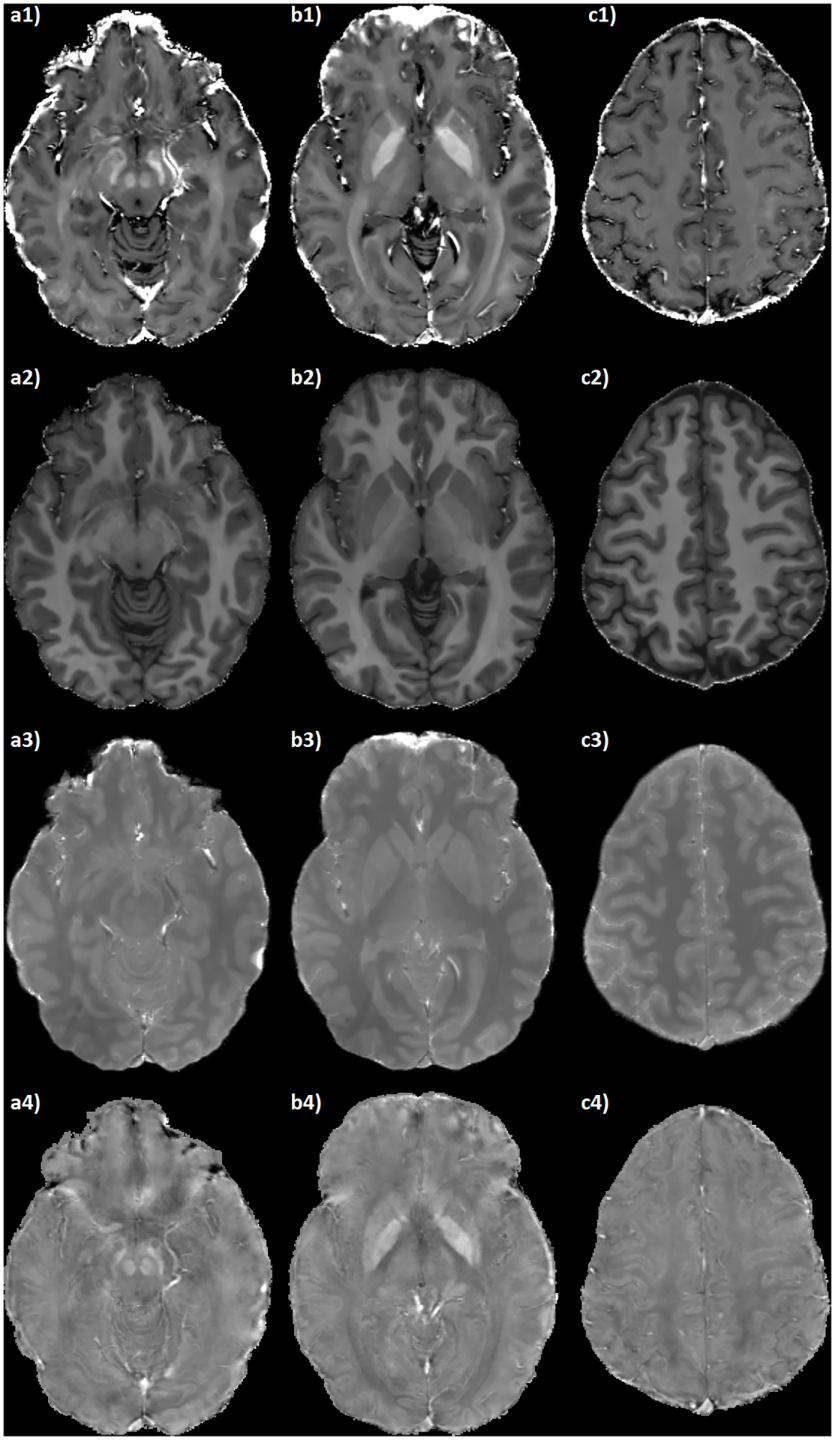
Axial RESUME maps of the brain. Slices at the level of midbrain (a), basal ganglia (b) and centra semiovalia (c) of: 1) $R_{2}^{*}$ map ([0 ∼ 50] s^−1^); 2) *R*_1_ map ([0 ∼ 2] s^−1^); 3) PD map ([0 ∼ 1] arbitrary units); 4) QSM ([−300 ∼ 300] ⋅ 10^−9^).

**Fig 5:**
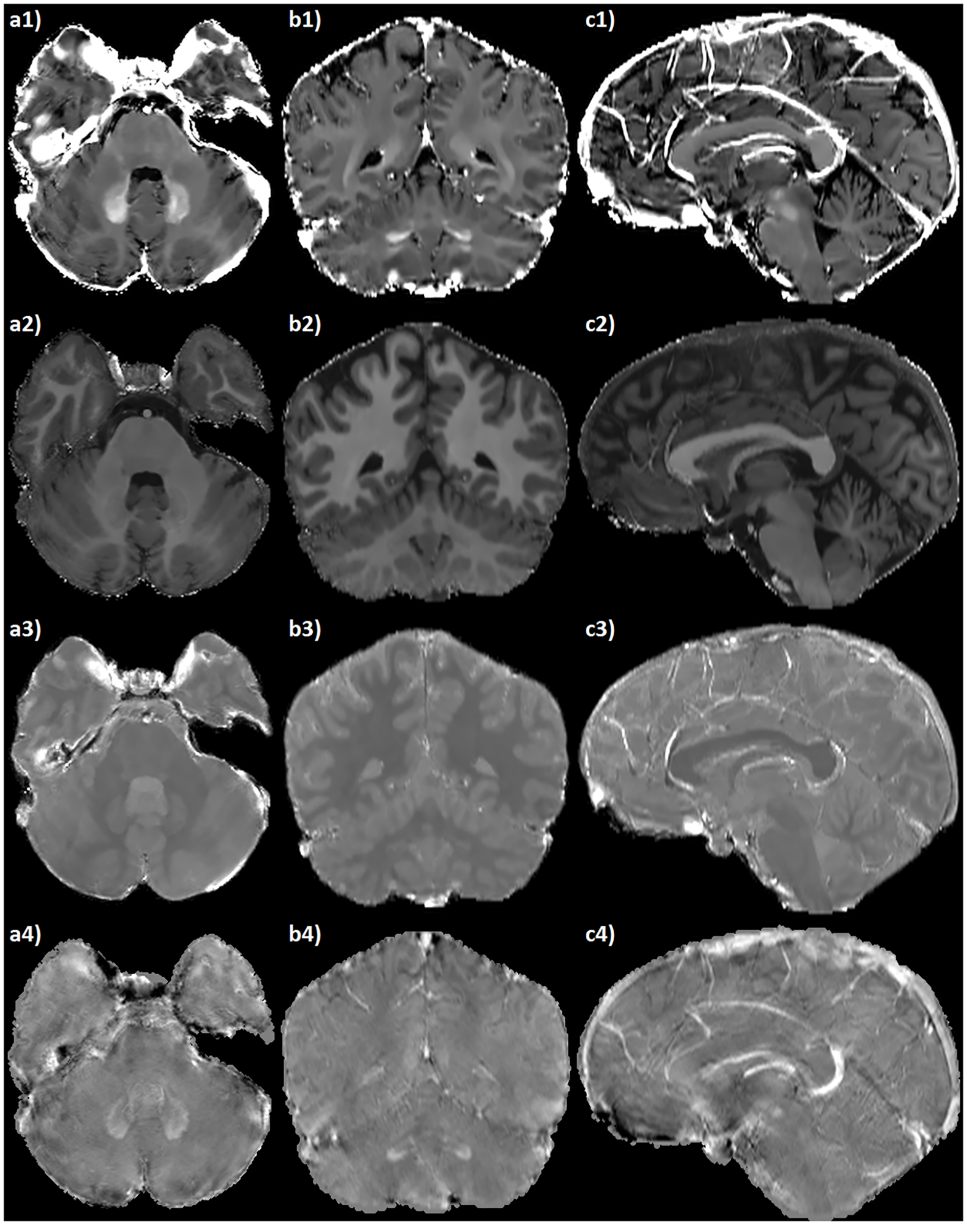
Multi-planar reconstructions of the RESUME maps. Axial (a), coronal (b) and sagittal (c) brain slices of: 1) $R_{2}^{*}$ map ([0 ∼ 50] s^−1^); 2) *R*_1_ map ([0 ∼ 2] s^−1^); 3) PD map ([0 ∼ 1] arbitrary units); 4) QSM ([−300 ∼ 300] ⋅ 10^−9^) maps. The axial and coronal slices are centered at the level of the cerebellar dentate nuclei (remarkably conspicuous in the $R_{2}^{*}$ and QSM maps due to their increased iron content); the sagittal slice is centered on the midline, where the deoxyhemoglobin-rich intracranial veins are particularly evident in the $R_{2}^{*}$ and QSM maps.

The derived maps exhibit a uniform quality throughout the entire FOV, and provide adequate spatial and contrast resolution for clear identification of the brain structures at different axial levels (see Fig 4). Moreover, the resolution of the acquired datasets allows for satisfactory multi-planar reconstructions (see Fig 5).

**Fig 6:**
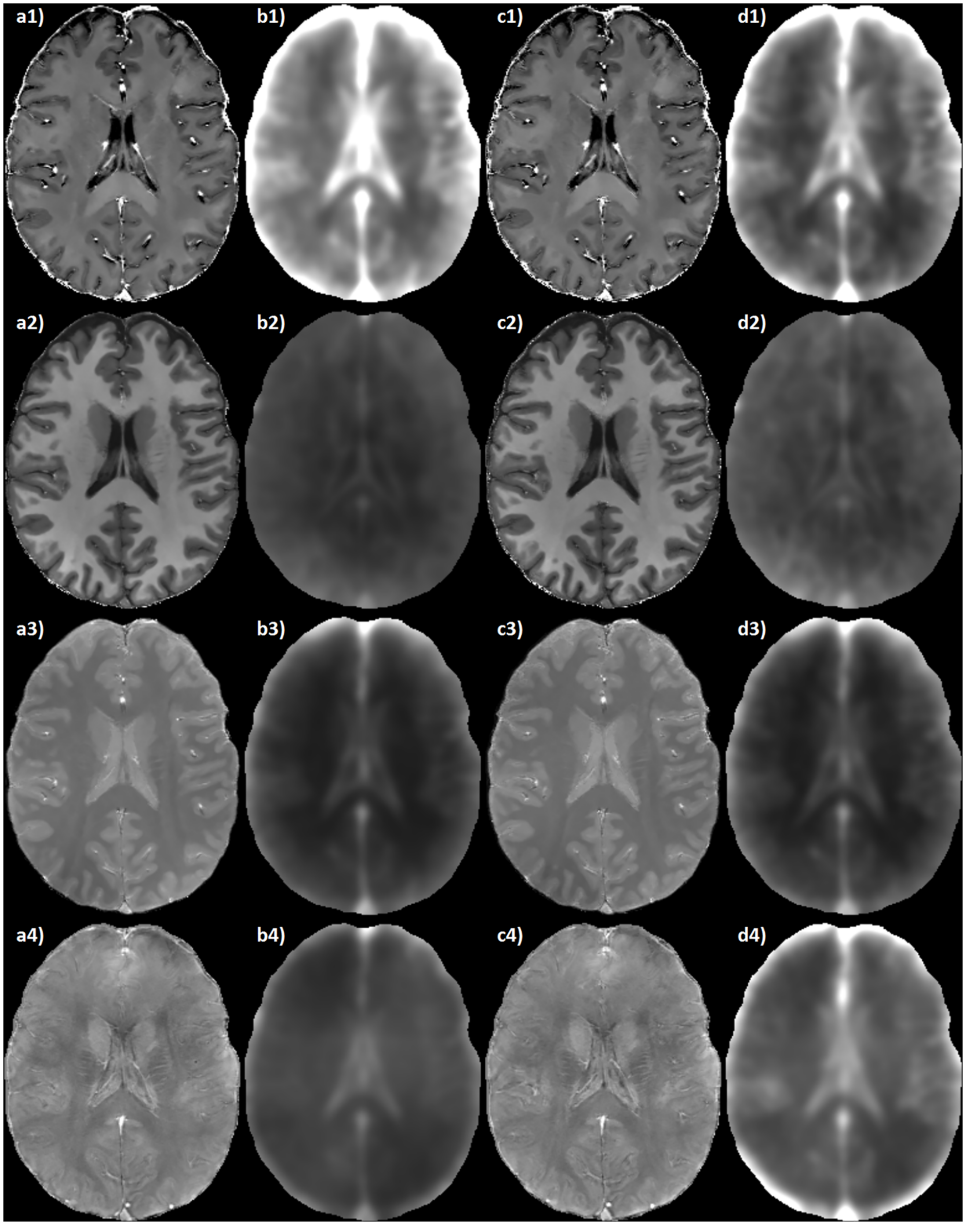
Reproducibility and accuracy of the RESUME maps. From left to right: a) RESUME maps of the spatial reference (HC1); b) voxelwise average, in the pool of subjects, of the $\left\{\sigma_{j}\left(\vec{x}\right)\right\}$ maps spatially normalized to HC1; c) qMRI reference maps of HC1 obtained following [16] (for $R_{2}^{*}$, *R*_1_ and PD maps) and from the 8-echo GRE (for the QSM); d) voxelwise average, in the pool of subjects, of the $\left\{\delta_{j}\left(\vec{x}\right)\right\}$ maps spatially normalized to HC1. From top to bottom: 1) $R_{2}^{*}$ map ([0 ∼ 50] s^−1^); 2) *R*_1_ map ([0 ∼ 2] s^−1^); 3) PD map ([0 ∼ 1] arbitrary units); 4) QSM ([−300 ∼ 300] ⋅ 10^−9^). Please note that the averages of $\left\{\sigma_{j}\left(\vec{x}\right)\right\}$ (b) and $\left\{\delta_{j}\left(\vec{x}\right)\right\}$ (d) are shown with an enhancement factor of 20.

Reproducibility and accuracy measures of RESUME were available both for visual inspection (see Fig 6) and in terms of summarizing numerical indices. In particular, in the pool of the analyzed subjects, the median values of $\left\{\sigma_{j}\left(\vec{x}\right)\right\}$ within the brain mask were normalized to the range of the associated $\left\{\mu_{j}\left(\vec{x}\right)\right\}$ maps, and their mean values were (1.38 ± 0.19)% for *R*_1_ map, (3.11 ± 0.82)% for $R_{2}^{*}$ map, (1.22 ± 0.20)% for PD map, and (1.43 ± 0.15)% for QSM. Similarly, the averages over the normalized median values of $\left\{\delta_{j}\left(\vec{x}\right)\right\}$ were (1.38 ± 0.17)% for *R*_1_ map, (2.15 ± 0.59)% for $R_{2}^{*}$ map, (1.17 ± 0.15)% for PD map, and (1.83 ± 0.23)% for QSM.

The RESUME values measured in the ROIs manually drawn in different brain structures of the HCs largely overlap with the corresponding ranges derived from the reference method [16] in the same cohort of subjects or found in the literature for different groups of HCs (see Table 1).

**Table 1:** Mean and standard deviation of the qMRI values in different brain regions, measured by RESUME or by the reference method [16] (Ref) in the healthy controls or found in the literature. PD values are normalized to the cerebrospinal fluid (CSF). Relaxometry values are compared with other 3D measures at 3 T.

	*R*_1_ (s^−1^)	$R_{2}^{*}$ (s^−1^)	PD (CSF)	QSM ⋅ 10^9^
White matter	1.08 ± 0.10	21.1 ± 1.1	0.718 ± 0.015	−5 ± 10
	1.07 ± 0.11 (Ref)	21.2 ± 1.2 (Ref)	0.699 ± 0.016 (Ref)	−2 ± 13 (Ref)
	1.036 ± 0.036 [39]	21.0 ± 0.8 [39]	0.742 ± 0.070 [39]	−18 ± 9 [40]
	1.190 ± 0.071 [41]		0.683 ± 0.006 [42]	
Cortical grey matter	0.624 ± 0.032	15.1 ± 1.6	0.852 ± 0.037	23 ± 30
	0.603 ± 0.045 (Ref)	14.6 ± 1.9 (Ref)	0.840 ± 0.043 (Ref)	21 ± 27 (Ref)
	0.609 ± 0.008 [39]	15.2 ± 0.4 [39]	0.796 ± 0.078 [39]	16 ± 10 [40]
	0.622 ± 0.043 [41]		0.844 ± 0.019 [42]	
Head of caudate nucleus	0.625 ± 0.054	20.2 ± 1.6	0.883 ± 0.019	68 ± 31
	0.615 ± 0.046 (Ref)	19.5 ± 1.4 (Ref)	0.860 ± 0.039 (Ref)	66 ± 29 (Ref)
	0.683 ± 0.022 [39]	18.2 ± 1.2 [39]	0.851 ± 0.084 [39]	60 ± 30 [43]
	0.719 ± 0.025 [41]		0.827 ± 0.016 [42]	78 ± 32 [44]
Putamen	0.663 ± 0.052	23.0 ± 2.0	0.886 ± 0.013	29 ± 16
	0.656 ± 0.051 (Ref)	22.8 ± 1.2 (Ref)	0.865 ± 0.018 (Ref)	30 ± 15 (Ref)
	0.752 ± 0.040 [41]	30.6 ± 4.7 [45]		70 ± 20 [40]
	0.70 ± 0.05 [46]			40 ± 20 [43]

**Fig 7:**
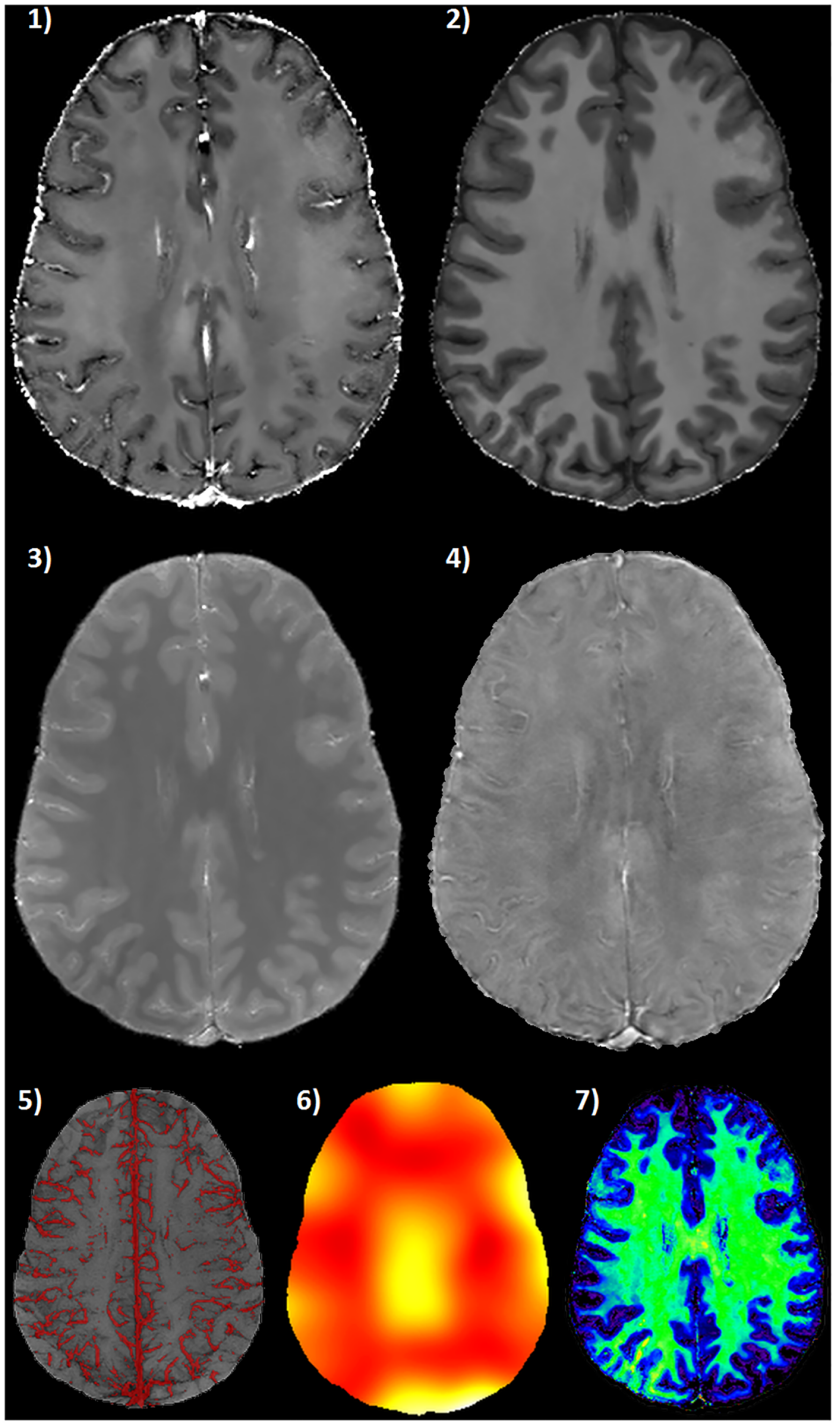
Extended qMRI processing of the RESUME protocol. Axial brain slices of: 1) $R_{2}^{*}$ map ([0 ∼ 50] s^−1^); 2) *R*_1_ map ([0 ∼ 2] s^−1^); 3) PD map ([0 ∼ 1] arbitrary units); 4) QSM ([−300 ∼ 300] ⋅ 10^−9^); 5) MAVEN vein segmentation; 6) OEF map ([0 ∼ 0.4]); 7) MTVF map ([0 ∼ 0.5]). MAVEN segmentation is Maximum-Intensity-Projected on a slab thickness of 20 mm and fused on the minimum-Intensity-Projection of the corresponding SWI slab (computed from the second echo of the dual-echo GRE), which serves as anatomical reference.

The output of an extended quantitative processing entirely based on the RESUME protocol is shown in Fig 7, where *R*_1_, $R_{2}^{*}$, PD and QSM come along with the MAVEN vein segmentation, the OEF and MTVF maps.

## Discussion

The search of quantitative and reproducible measures in MRI is of unquestionable interest for the scientific community. Indeed, qMRI methods directly focus on physical and objective parameters that could both increase the sensitivity of clinical MR scans, thus providing unique information about brain pathophysiology, and simplify the data harmonization in longitudinal or multicentric studies.

However, despite the growing interest of the neuroimaging community for qMRI (which provides hints on longitudinal and transverse relaxation processes, spin density, magnetic susceptibility, etc.), a certain skepticism usually creeps up at the moment of designing an acquisition protocol that could include it, for three main reasons.

First, the total acquisition time of a qMRI protocol that yields adequate resolution and SNR maps may be disheartening: in [47, 48], *R*_1_ and *R*_2_ maps (neglecting susceptibility phenomena and PD) were obtained in about 15 minutes, while the method described in [16] squeezed in the same duration a complete high-resolution qMRI scheme; conversely, other authors succeeded in lowering the acquisition time (in the order of 5 minutes), but they greatly sacrificed the resolution ([49, 50] stooped to a slice thickness of 5 mm).

Second, the sequences required by the great majority of the qMRI approaches are usually of poor radiological relevance as source of traditional MR image contrasts: in a clinical context, for instance, a *T*_2_-weighted Fast Spin Echo (FSE) is commonly preferred to the TrueFISP [16, 51, 52]; a Magnetization-Prepared RApid Gradient-Echo (MPRAGE) or *T*_1_-weighted SE are usually acquired instead of short *T*_*E*_-GRE; a PD-weighted FSE, if necessary, substitutes the low FA-GRE [42, 48]; etc.

Last but not least, the search for efficient or accurate qMRI protocols often leads to the development of prototype sequences [49, 50, 53] that are, as such, rarely available if not exclusive of the developing research center.

RESUME scheme reverses the situation, being based on a clinical sequence that leads to the widespread SWI processing, thus exploiting an acquisition time that is usually accorded in a protocol design. Given that the standard SWI reconstruction is provided, the RESUME approach allows for an additional reconstruction of a complete set of qMRI maps (*R*_1_, $R_{2}^{*}$, PD and QSM) at the cost of a 50% additional acquisition time (typically in the order of 2 minutes for high-resolution SWI).

The derived maps share the same resolution of the SWI, thus being suitable for multi-planar reconstructions (see Fig 5) and for the study of thin structures such as the cortex, small gray matter nuclei and medullary veins (see Figs 4 and 7).

Despite the short acquisition time (overall protocol duration of ∼ 7 minutes), the derived qMRI datasets exhibit a considerably high SNR even at a simple visual inspection. This is further confirmed by the confidence interval maps (see Fig 6), whose extent encompasses general reproducibility aspects besides the phenomena strictly connected to noise propagation.

A comparison with the maps obtained by an established state-of-the-art qMRI protocol [16] highlights an excellent accuracy of the RESUME results: indeed, the magnitude of the difference between the values estimated by the two methods falls within the limits fixed by the reproducibility issues. Moreover, the ranges of RESUME measures in different brain structures of HCs are consistent with those found in the literature for a variety of different qMRI approaches (see Table 1).

In this context, it is worth stressing the 3D nature of the acquired sequences that lead to the estimation of the relaxation parameters. Indeed, as most clearly pointed out in [32], relaxometry schemes based on 2D imaging sequences are prone to severe and barely rectifiable inaccuracies, due to the intrinsic intra-voxel FA variations of the slice-selective RF pulses. Furthermore, a comparison of Inversion Recovery (IR)-based *T*_1_-mapping schemes [41] showed that 2D multislice acquisitions, such as IR-FSE, lead to an underestimation of *T*_1_, due to several source of errors (such as flow, perfusion, through-plane motion, magnetization transfer caused by off-resonance excitation, non-ideal slice profiles, etc.) that are much less relevant on 3D sequences. On the other hand, relaxometry from 3D sequences can easily include *B*_1_-inhomogeneity correction schemes, *e*.*g*. based on the acquisition of the *B*_1_-map, and this seems consistent with the substantial agreement of the longitudinal relaxation rates measured in several brain structures by RESUME and by multiple MPRAGEs acquired at different inversion times [41].

From a computational point of view, the analytical RESUME solutions for the relaxometry equations are particularly beneficial for the processing execution time: the negligible burden associated with the computation of Eqs 4, 21 and 23 allows for an inexpensive application of the iterative *B*_1_-inhomogeneity correction adopted, so that the denoising step remains the bottleneck of an acceptable 10-minutes pipeline.

Finally, the interest in acquiring a thorough qMRI protocol like RESUME may also go beyond the extraction of classical relaxometry or susceptibility maps. In fact, the reconstructed parameters allow for additional quantitative processing steps that rely on the primary RESUME maps, such as the vessel-based analyses leading to vein segmentation and OEF maps, or the assessment of macromolecule density and local physico-chemical environment (see Fig 7).

In conclusion, the proposed acquisition and processing scheme allows for an accurate, reproducible and time-affordable strategy for obtaining different quantitative measures from a brain MRI scan. These features make RESUME feasible not only for research aims, but also for clinical practice, in light of an always increasing incorporation of qMRI protocols in the neuroradiological routine.
